# Qualitative Evaluation of a Health Literacy Program for Older Adults Who Live in a Community Dwelling in Brazil

**DOI:** 10.3928/24748307-20240722-01

**Published:** 2024-07

**Authors:** Andreivna Kharenine Serbim, Julie Ayre, Lisiane Manganelli Girardi Paskulin, Don Nutbeam, Danielle Muscat

## Abstract

To address current gaps in health literacy research and practice in low-resource settings, the 'Alfa-Health Program' was designed to improve health literacy in older adults who live in a community dwelling in a socioeconomically disadvantaged community in North-East Brazil. In this longitudinal qualitative study, participants were interviewed before and after participating in the group-based program that was delivered November 2017 to December 2017 in the Primary Care Health Unit. Semi-structured interviews were guided by a previously validated health literacy instrument, translated and adapted for use in Brazil. Data was analyzed using Framework analysis. Of the 21 participants, the majority were age 60 to 69 years with a median of 4-years of school education. Our analysis identified self-reported improvements in health knowledge, behaviors, and skills that matched program content and indicated that participants were supported to manage their health conditions more autonomously. Other themes reflect the distributed nature of health literacy and the potential for group-based health literacy programs to facilitate feelings of social support and cohesion through co-learning. However, age-related deficits in memory and external and structural factors remained important barriers to program participation. This study provides insight into developing health literacy in low-resource settings with older adults, where health literacy is compounded by social determinants and cognitive and sensory changes that contribute to health disparities. Although the targeted Alfa Health Program addresses calls to ensure that priority is proportionate to need by reaching and engaging population groups who are disproportionately affected by low health literacy, further work is needed to adapt the program for people who are unable to read or write. [***HLRP: Health Literacy Research and Practice*. 2024;8(3):e140–e150.**]

## Abstract (plain language summary)

**Plain Language Summary: **Our team developed a health literacy program for older adults living in Brazil. We explored the impact of the program by interviewing participants before and after the program. Participants reported improvements in health knowledge, behaviors, and skills and reflected on feelings of social support that they received from the program. However, difficulties with memory and challenges getting to the program were important barriers to participation.

Health literacy is conceptualized as the personal skills and competencies which determine the ability of individuals to gain access to, understand, and use information in ways that promote and maintain good health (Nutbeam & Muscat, 2021). To be health-literate means not only having access to health information and knowledge but also being able to find, process, appraise and understand it as a crucial step to managing health (Matas et al., 2018). Health literacy is a type of power that is critical for self-determination and a tool for creating personal, family, organizational, and societal change (Paasche-Orlow et al., 2018).

Unsurprisingly, health literacy is one of the strategies noted in efforts to provide better lives for all citizens (Flecha et al., 2011). It has long been proposed as a measurable outcome of health education, and as a way of measuring educational impact that fits into a broad model of health promotion (Nutbeam, 2021). As such, one way to improve health literacy is through structured education programs. Such programs can be distinguished by their focus on skills to support health literacy rather than on a transfer of health information (Rudd, 2002). This focus seeks to equip people to make a range of more autonomous decisions relating to their health, to adapt their decision-making to changing contexts and personal circumstances, and to gain a broader understanding of health and its determinants (Nutbeam, 2021). Structured health literacy programs can bring people together, offering the opportunity to share knowledge and experiences, and create common understandings related to health (de Wit et al., 2018; Rudd, 2002). Evidence suggests that structured education interventions can help develop health literacy skills and improve some health behaviors (Nutbeam et al., 2018; McCaffery et al., 2019; Muscat et al., 2019). However, participatory programs and experiential learning are still not the norm in either heath or adult education settings (Rudd, 2002).

There are added challenges related to chronic diseases and cognitive and sensory changes that contribute to decreased health literacy in older adults (Chesser et al., 2016). Even in healthy adults, research suggests that health literacy scores decrease with age (Wolf & Bailey, 2012). Developing health literacy through education aligns with the World Health Organization's (WHO) recommendation for lifelong learning to help people develop skills and confidence in health decision-making across the life-course (WHO, 2002). However, there are a few interventions for older people to improve health literacy. A systematic review of theory-based interventions aimed at promoting e-health literacy among older adults, for example, identified only 12 studies globally (Pourrazavi et al., 2020). This represents an important gap as older people face additional and specific barriers to developing and maintaining their health literacy skills.

As well as there being research gaps related to age, little research has been undertaken outside of Western (predominantly English-speaking) countries or with vulnerable communities. For example, to date, relatively little research into health literacy has been undertaken in Brazil. The small number of Brazilian studies that have been conducted consistently indicate that a significant majority of older adults in Brazil have limited health literacy, assessed through a variety of measurement instruments. Estimates range from 52% to 74% (Apolinário et al., 2012; Santos & Portella, 2016). More recently, a qualitative analysis identified that older adult Brazilians living in socially deprived geographical regions experience difficulties accessing, understanding and communicating health information, often in the context of chronic disease. At the most functional level, few participants demonstrated an understanding of their health conditions and indicated that they often did not understand medical terms and did not actively seek health information (Serbim et al., 2022). Here, systemic barriers prevent people without educational privilege from fulfilling their health goals; health and illness (and health disparities) are largely determined by the maldistribution of social and environmental forces and exposures—problems that can be addressed, at least in part, through enhancing health literacy (Paasche-Orlow et al., 2018).

We developed an education-based health literacy intervention (the ‘Alfa-Health Program’) to improve health literacy in older populations accessing primary care in Brazil. Feasibility testing indicated that the intervention achieved relatively high levels of participation, positive feedback from participants, some positive (although not statistically significant) increases in health literacy and some significant changes in health behaviors, such as vaccination (Serbim et al., 2020). The current manuscript presents the findings from the qualitative evaluation of this health literacy program among older adults who live in a community dwelling in Brazil. The purpose of this evaluation was to explore the perceptions of changes in health and health literacy after participating in the program and to elucidate any other perceived impacts in a disadvantaged community in North-East Brazil.

## Methods

### Study Design

This is a longitudinal qualitative study in which participants were interviewed before and after participating a health literacy training intervention. The health literacy intervention was an educational program (the ‘Alfa-Health Program’) designed to improve critical health literacy in older population in a primary care health unit (PHCU) in Brazil (Serbim et al., 2020).

### Setting

The study took place at one PHCU in the city of Arapiraca, Brazil. Arapiraca is a city in the countryside of the Northeast of Brazil, with approximately 230,000 people and the Human Development Index of 0.649 (United Nations, 2011). There are differences between geographical areas in Brazil and the Northeast is one of the regions with the lowest levels of health, education and economic development. Brazil's Unified Health System (Sistema Único de Saúde) has been widely acknowledged as an example of successful health system reform in Latin America and has made good progress towards achieving Universal Health Coverage (Massuda et al., 2018). The PHCU selected for the study was located in a suburb of Arapiraca with relatively high poverty and social vulnerability, including high rates of violence.

### Intervention

The Alfa-Health Program is an educational program designed to improve health literacy among older adults. The focus was on developing transferable skills that equip people to make a range of more autonomous decisions relating to their health, and to adapt to changing circumstances. The intervention was conducted by a nurse (A.K.S.) over a 20-week period through weekly meetings in a group room in the PHCU. The main topics focused on access to the health system; safe use of medicines; healthy lifestyle choices; the use of prevention services; mental health; men's and women's health; human rights; social participation; retirement; and healthy environments. The topics were based on the pillars (health, lifelong learning, participation and safety) of the Active Aging Policy (WHO, 2002), and materials available from the Brazilian Ministry of Health (Ministry of Health, Brazil, 2017).

The program was underpinned by problematizing pedagogy (Freire, 1998) to enable a more participatory health educational practice. This approach was selected given the relationship between health promotion, community and personal empowerment, where the development of skills and attitudes are seen to act in favor of health. Older adult participants' own knowledge was considered the starting point for the pedagogical process to enable the construction of knowledge based on respect for the autonomy and appreciation of the creativity of program participants (Lucena et al., 2016). Together with the technical-scientific knowledge of the nurse educator, this approach was intended to contribute to the multiplication of knowledge. Based on our pedagogical approach, at the beginning of each meeting, participants shared what they already knew about the main health topics, their feelings toward the topic and had the opportunity to ask questions. Educational background, participant lay/popular knowledge and previous experiences were also considered as important factors that influenced abilities to seek and understand health information (Jordan et al., 2010).

### Participants and Recruitment

The first researcher (A.K.S.), with the assistance of the research team and the professionals of the health unit, invited the older adults to participate in the study. Initial contact was made by phone call or in person (at the PHCU or by home visit) to assess for eligibility. Inclusion criteria for the study were: (1) older than age 60 years, (2) enrolled in the PHCU, and (3) self-reported ability to read and write Portuguese, the official language in Brazil. The exclusion criteria were self-reported inability to read or write in Portuguese (as indicated in the PHCU register system); inability to respond to an interview (e.g. hearing or vision problems); cognitive impairment, assessed using a score <6 on a 10-point cognitive screener (Apolinário et al., 2016); or participation in an existing health promotion program (such as for diabetes, hypertension or similar).

All participants eligible for the Alfa-Health Program were invited by the first author (A.K.S.) to participate in a qualitative interview before the program commenced. After the 20-week intervention period, all remaining participants were again invited to participate in a second interview.

### Data Collection

In total, 21 individual interviews (approximately 60 minutes each) were conducted at baseline prior to program commencement from November 2017 to December 2017. This represented all older adults enrolled in the program. Thirteen interviews were conducted at follow-up after the intervention period (June 2018 and July 2018). Throughout the intervention period, 8 participants withdrew from the program (1 accident; 2 moved; 5 withdrew from the intervention). Of the 13 participants enrolled at the end of the intervention period, all agreed to participate in the qualitative interview. All interviews were conducted by the first author in a room at the PHCU, audio-recorded and transcribed verbatim in Portuguese. Transcripts were then translated into English for data analysis.

For data collection, participants initially were asked to provide demographic information including sex, age, schooling, income and housing. Interviews were then guided by a qualitative health literacy instrument composed of open-ended and closed-ended questions, developed by Canadian researchers and translated and adapted for use in Brazil (Paskulin et al., 2011). The questions start by asking the participants to choose an area of health that interests or concerns them and talk about their experiences relating to that topic. They are specifically asked about accessing, understanding and communicating health information, as well as the impact of health information on their lives (Begoray & Kwan, 2012; Paskulin et al., 2012). For the purposes of this study, participants did not necessarily need to select the same health concern for both interviews (i.e. pre- and post-intervention).

To assess perceived impact at the end of the intervention program, participants were also asked an open-ended question: “What difference did the Alfa-Health Program make in your life?”

### Data Analysis

Qualitative Solutions Research Nvivo software (QSR Nvivo) version 11.0 was used to organize the qualitative data. Data were then analyzed according to the five key steps of Framework analysis (Ritchie et al., 2003), a matrix-based approach to thematic analysis. A description of each analytic stage is presented in **Table 1**.

**Table 1:**
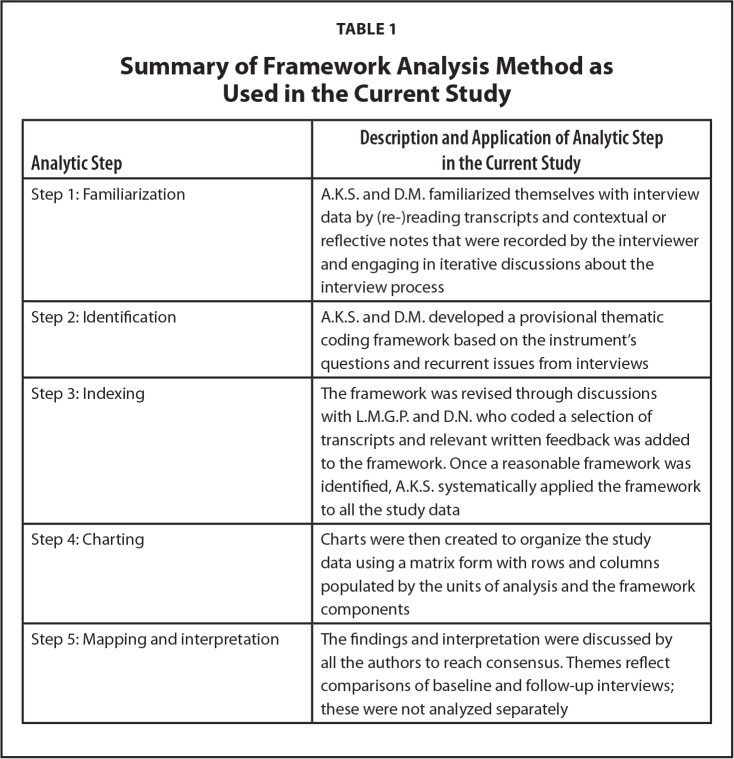
Summary of Framework Analysis Method as Used in the Current Study

**Analytic Step**	**Description and Application of Analytic Step in the Current Study**
Step 1: Familiarization	A.K.S. and D.M. familiarized themselves with interview data by (re-)reading transcripts and contextual or reflective notes that were recorded by the interviewer and engaging in iterative discussions about the interview process
Step 2: Identification	A.K.S. and D.M. developed a provisional thematic coding framework based on the instrument's questions and recurrent issues from interviews
Step 3: Indexing	The framework was revised through discussions with L.M.G.P. and D.N. who coded a selection of transcripts and relevant written feedback was added to the framework. Once a reasonable framework was identified, A.K.S. systematically applied the framework to all the study data
Step 4: Charting	Charts were then created to organize the study data using a matrix form with rows and columns populated by the units of analysis and the framework components
Step 5: Mapping and interpretation	The findings and interpretation were discussed by all the authors to reach consensus. Themes reflect comparisons of baseline and follow-up interviews; these were not analyzed separately

### Ethical Considerations

This study was approved by Federal University of Rio Grande do Sul Human Research Ethics Committee (Nº CAAE: 72106817.2.0000.5347)/Brazil. All participants provided written informed consent.

## Results

**Table 2** shows that among the 21 participants who participated in the first interview, 14 were women and the majority (*n* = 15) were between ages 60 and 69 years. Of the total number, 11 were married and 6 were widowed. Most reported that they lived with family members (*n* = 16). Participants had a median of 4 years of education over the course of their lives, and all received the minimum state pension ($191.00 per month) as their only income.

**Table 2:**
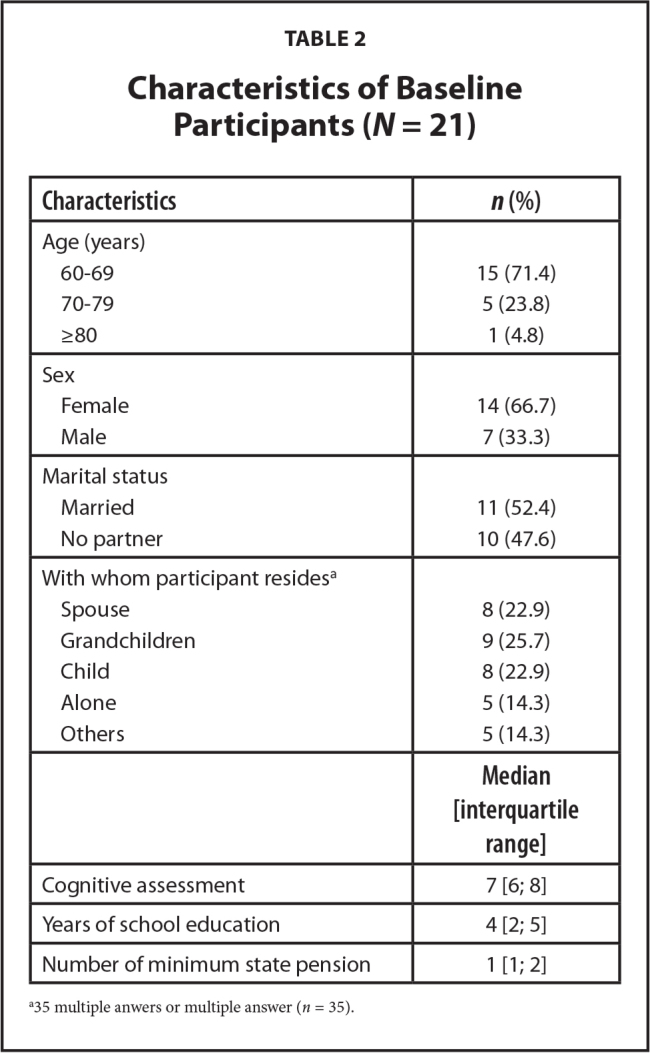
Characteristics of Baseline Participants (*N* = 21)

**Characteristics**	***n* (%)**

Age (years)	
60–69	15 (71.4)
70–79	5 (23.8)
≥80	1 (4.8)

Sex	
Female	14 (66.7)
Male	7 (33.3)

Marital status	
Married	11 (52.4)
No partner	10 (47.6)

With whom participant resides^a^	
Spouse	8 (22.9)
Grandchildren	9 (25.7)
Child	8 (22.9)
Alone	5 (14.3)
Others	5 (14.3)

	**Median [interquartile range]**

Cognitive assessment	7 [6; 8]

Years of school education	4 [2; 5]

Number of minimum state pension	1 [1; 2]

a 35 multiple anwers or multiple answer (*n* = 35).

The health concerns chosen by participants at baseline were varied and included diabetes mellitus (*n* = 4), osteoporosis (*n* = 3), problems in the back (*n* = 3), problems in the prostate (*n* = 2) and gastritis (*n* = 2), varicose veins (*n* = 2), tiredness (*n* = 2), dizziness (*n* = 1), insomnia (*n* = 1) and glaucoma (*n* = 1). At follow-up, the health concerns chosen were arterial hypertension (*n* = 4), diabetes mellitus (*n* = 3), osteoporosis (*n* = 2), alcoholism (*n* = 1), cataracts (*n* = 1), gastritis (*n* = 1) and anemia (*n* = 1).

Themes identified from the qualitative analysis included: (1) impact of the Alfa-Health Program; (2) social networks and distributed literacy, and (3) barriers to engagement and implementation.

### Impact of the Alfa-Health Program

***Knowledge and control.*** The impact of the Alfa-Health program was revealed both in participants' explicit narratives, as well as in the changes in the way in which discussed their chosen health concern(s) from the first interview (prior to the program) to the final interview after program completion (**Table 3**). Most obviously, most participants explicitly stated that they had acquired additional basic knowledge about health from the Alfa-Health program and felt more confident understanding health information (**Table 3**; Quote 1a). Often, such reflections were couched in an acknowledgement that such knowledge gains occurred despite participants' relative lack of education and other perceived age-related deficits (e.g., “vision problem[s]” – Participant 7, female, 66 years).

**Table 3:**
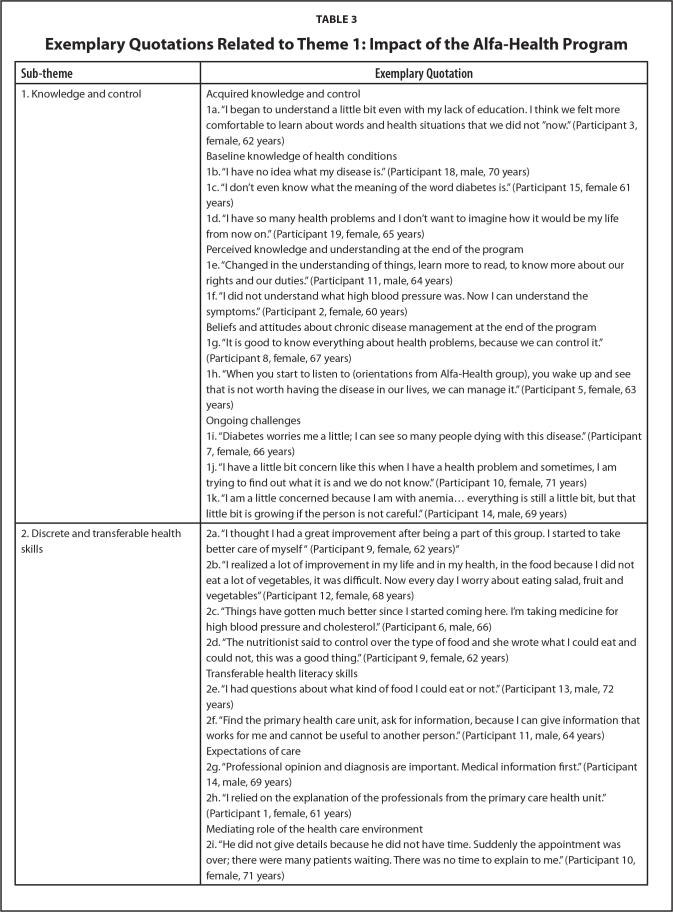
Exemplary Quotations Related to Theme 1: Impact of the Alfa-Health Program

**Sub-theme**	**Exemplary Quotation**

1. Knowledge and control	Acquired knowledge and control
	1a. “I began to understand a little bit even with my lack of education. I think we felt more comfortable to learn about words and health situations that we did not ”now.” (Participant 3, female, 62 years)
	Baseline knowledge of health conditions
	1b. “I have no idea what my disease is.” (Participant 18, male, 70 years)
	1c. “I don't even know what the meaning of the word diabetes is.” (Participant 15, female 61 years)
	1d. “I have so many health problems and I don't want to imagine how it would be my life from now on.” (Participant 19, female, 65 years)
	Perceived knowledge and understanding at the end of the program
	1e. “Changed in the understanding of things, learn more to read, to know more about our rights and our duties.” (Participant 11, male, 64 years)
	1f. “I did not understand what high blood pressure was. Now I can understand the symptoms.” (Participant 2, female, 60 years)
	Beliefs and attitudes about chronic disease management at the end of the program
	1g. “It is good to know everything about health problems, because we can control it.” (Participant 8, female, 67 years)
	1h. “When you start to listen to (orientations from Alfa-Health group), you wake up and see that is not worth having the disease in our lives, we can manage it.” (Participant 5, female, 63 years)
	Ongoing challenges
	1i. “Diabetes worries me a little; I can see so many people dying with this disease.” (Participant 7, female, 66 years)
	1j. “I have a little bit concern like this when I have a health problem and sometimes, I am trying to find out what it is and we do not know.” (Participant 10, female, 71 years)
	1k. “I am a little concerned because I am with anemia… everything is still a little bit, but that little bit is growing if the person is not careful.” (Participant 14, male, 69 years)

2. Discrete and transferable health skills	2a. “I thought I had a great improvement after being a part of this group. I started to take better care of myself “ (Participant 9, female, 62 years)“
	2b. “I realized a lot of improvement in my life and in my health, in the food because I did not eat a lot of vegetables, it was difficult. Now every day I worry about eating salad, fruit and vegetables” (Participant 12, female, 68 years)
	2c. “Things have gotten much better since I started coming here. I'm taking medicine for high blood pressure and cholesterol.” (Participant 6, male, 66)
	2d. “The nutritionist said to control over the type of food and she wrote what I could eat and could not, this was a good thing.” (Participant 9, female, 62 years)
	Transferable health literacy skills
	2e. “I had questions about what kind of food I could eat or not.” (Participant 13, male, 72 years)
	2f. “Find the primary health care unit, ask for information, because I can give information that works for me and cannot be useful to another person.” (Participant 11, male, 64 years)
	Expectations of care
	2g. “Professional opinion and diagnosis are important. Medical information first.” (Participant 14, male, 69 years)
	2h. “I relied on the explanation of the professionals from the primary care health unit.” (Participant 1, female, 61 years)
	Mediating role of the health care environment
	2i. “He did not give details because he did not have time. Suddenly the appointment was over; there were many patients waiting. There was no time to explain to me.” (Participant 10, female, 71 years)

Knowledge gains were also reflected in the way in which participants spoke about their chosen health concern(s). Participants at baseline appeared to have limited knowledge to explain their health conditions. Some expressed that they did not know much about the health condition(s) that they were living with or could describe the health problem in a general sense but were not confident describing the details (Quotes 1b and 1c). During the first interviews (before the Alfa-health Program), older adults also often focused on the symptoms or issues that generated discomfort in their lives and expressed negative feelings about aging, particularly in the context of living with multiple morbidities (Quote 1d). This contrasted discussions at the end of the program where participants' narratives about disease demonstrated greater knowledge and awareness, with the program having reported to have “opened our eyes more” (Participant 1, female, 61). Participants often explicitly stated that they perceived their health knowledge and understanding to have changed, expressed feeling more comfortable and confident to talk about health conditions and tried to explain their diseases in their own way (Quotes 1e and 1f).

Beliefs and attitudes about chronic disease management also appeared to have shifted to a discourse of greater 'control', with a focus on living well with and despite chronic disease (Quote 1g). The language used after the program seemed to be more positive and optimistic, with several older adults expressing greater confidence in relation to self-management (Quote 1h).

Despite this perceived change in orientation, participants did still express concerns about their ability to manage some health conditions, and ongoing challenges in obtaining health information (Quotes 1i+1j). Descriptions of health conditions—including their etiology: T and progression—remained limited in some instances despite having participated in the program (Quote 1k).

***Discrete and transferable health skills.*** After the Alfa-Health Program, participants reflected on changes to health habits stemming from their participation (**Table 3**; Quote 2a). This included changes in relation to taking medication and to healthy eating (Quotes 2b and 2c). In this context, participants acknowledged that they had to “do things right” (Participant 10, female, 71) to support their health in older age. Notably, these changes were often couched in more general discussions about other forms of care received through the PHCU, making it difficult to disentangle the discrete impact of the Alfa Health program (Quote 2d).

**Table 4:**
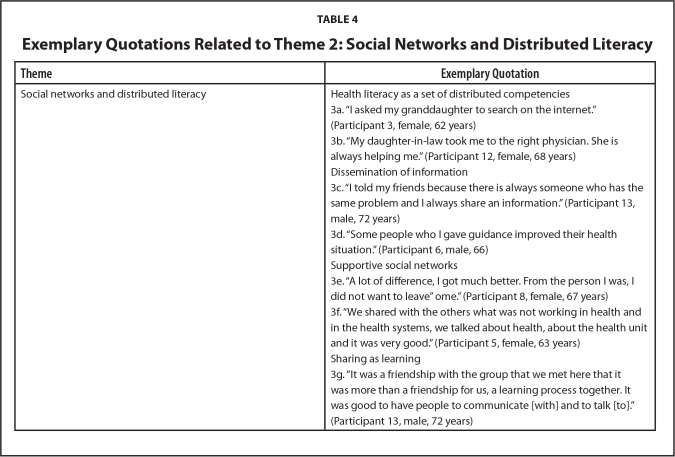
Exemplary Quotations Related to Theme 2: Social Networks and Distributed Literacy

**Theme**	**Exemplary Quotation**

Social networks and distributed literacy	Health literacy as a set of distributed competencies
	3a. “I asked my granddaughter to search on the internet.” (Participant 3, female, 62 years)
	3b. “My daughter-in-law took me to the right physician. She is always helping me.” (Participant 12, female, 68 years)
	Dissemination of information
	3c. “I told my friends because there is always someone who has the same problem and I always share an information.” (Participant 13, male, 72 years)
	3d. “Some people who I gave guidance improved their health situation.” (Participant 6, male, 66)
	Supportive social networks
	3e. “A lot of difference, I got much better. From the person I was, I did not want to leave” ome.” (Participant 8, female, 67 years)
	3f. “We shared with the others what was not working in health and in the health systems, we talked about health, about the health unit and it was very good.” (Participant 5, female, 63 years)
	Sharing as learning
	3g. “It was a friendship with the group that we met here that it was more than a friendship for us, a learning process together. It was good to have people to communicate [with] and to talk [to].” (Participant 13, male, 72 years)

As well as reporting changes in health knowledge and behaviors, after taking part in the program there were also indications of improvements in more transferable health literacy skills. For example, participants reported asking more questions about their health situation to their healthcare providers (e.g. doubts about food, elderly dependence, solving health issues, understanding the complexity of disease management) and spoke more about the importance and value of individualized health information (Quotes 2e and 2f). This occurred in a context in which older adults often acknowledged that they had previously been “accustomed to not receive information” (Participant 20, female, 64 years) about their health.

Participants' narratives reflected more positive expectations regarding professional care and suggested that they were more aware of the importance of healthcare providers in providing trustworthy information (Quotes 2g and 2h). Although health care providers were seen as trustworthy sources of information, participants described continuing external and structural barriers to implementing the knowledge and skills learnt throughout the program. Health care encounters, for example, were still described as being time-limited and physician-led by participants after having completed the Alfa-Health program, limiting the extent to which older adults could obtain and clarify information about their health (Quote 2i). In this way, the further development of health literacy skills continued to be mediated by the health environments in which older adults found themselves.

***Social networks and distributed literacy.*** Many participants spoke about social aspects of the program and of their lives more generally. Such narratives reflected the distributed nature of health literacy. For example, it became evident that participants at baseline primarily relied on family and friends to find health information and access health services with and for them. In this way, the health literacy skills of “granddaughters,” “daughters-in-law,” and others supplemented or compensated for the (real or perceived) individual health literacy deficits of older adults. Health literacy here was a set of 'distributed competencies' which could be found dispersed through the individual's social network, rather than an exclusively individual attribute, as **Table 4** shows (Quotes 3a and 3b).

**Table 5:**
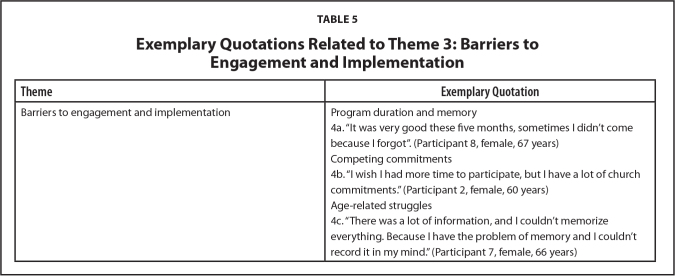
Exemplary Quotations Related to Theme 3: Barriers to Engagement and Implementation

**Theme**	**Exemplary Quotation**

Barriers to engagement and implementation	Program duration and memory
	4a. “It was very good these five months, sometimes I didn't come because I forgot”. (Participant 8, female, 67 years)
	Competing commitments
	4b. “I wish I had more time to participate, but I have a lot of church commitments.” (Participant 2, female, 60 years)
	Age-related struggles
	4c. “There was a lot of information, and I couldn't memorize everything. Because I have the problem of memory and I couldn't record it in my mind.” (Participant 7, female, 66 years)

After taking part in the Alfa-Health program, participants similarly discussed how they had shared or intended to share information through their social network and disseminate information to other people, such as friends and neighbors (Quotes 3c and 3d). Supportive social networks were also discussed within the context of the program itself. Participants consistently identified the benefits of social support from belonging to the Alfa-Health group. For many, this was expressed in a context in which they previously rarely left their homes or had wanted to. Such reflections situated the program as a beneficial yet unique space where older adults could come together (Quotes 3e and 3f).

Social cohesion within the group appeared to be facilitated by classroom practices which enabled the sharing of health information and experiences. Many participants, for example, found commonalities in shared health experiences and challenges which brought the group together and this was perceived to have facilitated learning (Quote 3g).

***Barriers to engagement and implementation.*** Despite generally positive attitudes towards the program, participants' interviews identified a variety of individual, environmental, and community-level barriers to participation. Absences were frequent across the 5 month intervention period, mainly on rainy or very hot days. Although participants lived close to the health unit, precarious conditions of infrastructure and vulnerability of the neighborhood (lack of transport, poor quality pavement and walking conditions, and concerns with personal safety) reduced the frequency of participation in the intervention. The long duration of the intervention was also noted in the interviews, with some participants reporting that they forgot to attend sessions over that period, as **Table 5** shows (Quote 4a).

Others reported the lack of time to participate in the intervention. Although the majority were retired, they had a lot of commitments with their family and especially with religious activities (Quote 4b). By and large, such accounts were marked by statements of “regret” (Participant 12, female, 68 years) for not having been able to attend every meeting. In addition to this, participants spoke of their substantial age-related struggles which impacted engagement with the program and retention of content. For example, a few participants discussed how poor memory contributed to missing meetings and being unable to retain the volume of information exchanged throughout the program (Quote 4c).

## Discussion

This manuscript presents the findings from the qualitative evaluation of the Alfa-Health health literacy program delivered to older adults who live in a community dwelling in Brazil in a socially deprived region of the country. Our analysis identified self-reported improvements in health knowledge, behaviors and skills which matched the content of the program and suggested that older adults were supported to manage their health conditions more autonomously. Other themes reflect the distributed nature of health literacy and the potential for group-based health literacy programs to facilitate feelings of social support and cohesion. However, age-related deficits in memory, competing commitments and external and structural factors remained important barriers to program participation and implementation of some health skills and behaviors.

The Alfa-Health program was based on a health literacy intervention delivered in established adult education settings in Australia (McCaffery et al., 2019). The findings from this Brazilian study bare similarities to the qualitative evaluation in Australia, including reports of improved knowledge and skills to engage in clinical encounters and manage health issues (Morony et al., 2018). Evaluation of a related intervention study in Japan (Ishikawa et al., 2018) also suggested that participants had a more realistic understanding and positive perception of healthcare providers, and a less passive relationship with the physician following participation. However, there are also some important differences related to applying this intervention in a low-resource primary care setting and with older adults who were not already enrolled in established educational programs. Our qualitative evaluation suggested that challenges to participation may have been exacerbated by external factors (lack of transport and concerns about personal safety, for example) and with older adults who reported forgetting to attend the program. In addition, the application of a broad-based health literacy program covering a range of topics posed additional challenges for older adults who were often unable to retain the amount of content covered over the 5-month intervention period. Although participants reported improvements in knowledge and health behaviors, their narratives related to specific health conditions identified remaining gaps in understanding. This feedback indicates that there would be benefit in developing shorter, more focused health literacy programs directed towards building skills and knowledge of specific health conditions for older adults in primary health settings. Additional communication methods may also be needed to support retention of health information and skills among adults with very low levels of literacy, tailored to older adults' preferred learning styles (Brooks et al., 2017).

This study also contributes to emerging evidence related to the important role social support and co-learning in health literacy interventions. A recommended approach to building trust and supporting health literacy development is through collaborative learning, or co-learning. Co-learning usually involves creating a learning environment where learners can interact and exchange health knowledge and experiences with family, community members or peers alongside health care professionals to learn from each other (de Wit et al., 2018). The creation of these open spaces has an important impact on the development of cohesive communities in which people feel comfortable and involved (Flecha et al., 2011). This is particularly important in contexts where there is poverty and social exclusion (Flecha et al., 2011). Our learners expressed the importance of social networks in the context of the Alfa-Health program—participants in this study reported receiving emotional (e.g. sharing experiences) and informational (e.g. advice and information) support from their peers and facilitators. However, other types of support identified by de Wit et al. (2018)—such as appraisal (e.g. information for self-evaluation) support—were less frequently mentioned. Further work is needed to understand why this is the case, and how this can be addressed in future interventions.

Outside of the program itself, our findings revealed that family was an important source of information and support for individuals, with family members often supplementing older adults' health literacy skills (e.g. by conducting internet searches for them; helping them to access health services). Participants in the intervention similarly shared health information that they received with the community. This is consistent with findings from other studies including a seminal longitudinal study of ‘distributed health literacy’ conducted in the United Kingdom which observed friends, family and colleagues as mediating the development and practice of health literacy by sharing knowledge, facilitating learning, contributing their own skills and supporting decision making (Edwards et al., 2012). Other studies have also depicted health information seeking as a collective process taking place in informal networks (Dutta et al., 2018; Muscat et al., 2022). This suggests that although only a small number of participants took part in the Alfa-Health Program, the impact may be magnified when they share what they have learned with their community. This has also been reported in previous studies (e.g., Morony et al., 2018; Flecha et al., 2011). However, more sophisticated methods for capturing the actual transfer of health literacy knowledge and skills in real world settings is needed beyond qualitative, self-report methodologies.

## Study Limitations and Strengths

Our study has both strengths and limitations. The exploratory, in-depth nature of this study enabled us to gain a broader understanding of program impacts from the perspective of participants, including impacts that are more difficult to measure (e.g., social connections) or not typically captured in quantitative evaluations of effectiveness. However, a limitation was that, although 21 participants who participated in the first interview, only 13 participated in the second interview after program implementation. As such, the qualitative data presented may reflect the opinions of those most engaged with the intervention and/or who were receiving benefit from the program. We may have missed the experiences of those who did not find the program useful.

This limitation is compounded by the nature of “researcher–researched relationship” (Berger, 2015) in this study in which the same member of the research team involved in delivering the intervention also conducted the interviews with participants and led the analysis of data. Although this ‘shared experience’ equipped the researcher with insights and sensitized them to certain dimensions of the data, it likely impacted the information that participants were willing to share, potentially shaping more positive evaluations of the program. To address this in data analysis, co-researchers were constantly consulted to reflect on the accuracy of the analysis, with iterative reflexive discussions about ways to mitigate the imposition of the teams' own values, beliefs, and perceptions about the program; and projection of biases (Berger, 2015).

Finally, participants in this study were also more literate than others in their community, by virtue of the intervention's literacy inclusion criteria. This means, for example, that findings do not reflect the experiences of people who cannot read and write in Portuguese. Future work is needed to develop health education activities/interventions that are accessible for these groups.

This study provides qualitative evidence that literate older adults in vulnerable communities in Brazil can develop key health literacy skills/confidence to improve their ability to access, understand and act on healthy information, and engage with health professionals. Participants reported positive perceptions of the program and appreciated the content as well as the social aspect of the program. An additional benefit was that they reported disseminating what they had learned to others in their community. The study also revealed some barriers to the development of health literacy among older adults in low-resource settings, such as barriers related to changes in aging. Further work is needed to test the effects of the intervention more systematically/quantitatively, address issues to improve retention, and to develop programs for other vulnerable groups, such as older adults who are unable to read and write.
